# Resistance Mechanisms to Antimicrobial Peptides in Gram-Positive Bacteria

**DOI:** 10.3389/fmicb.2020.593215

**Published:** 2020-10-21

**Authors:** Lucas Assoni, Barbara Milani, Marianna Ribeiro Carvalho, Lucas Natanael Nepomuceno, Natalha Tedeschi Waz, Maria Eduarda Souza Guerra, Thiago Rojas Converso, Michelle Darrieux

**Affiliations:** Laboratório de Biologia Molecular de Microrganismos, Universidade São Francisco, Bragança Paulista, Brazil

**Keywords:** antimicrobial peptides, gram-positive, AMP, resistance, streptococci

## Abstract

With the alarming increase of infections caused by pathogenic multidrug-resistant bacteria over the last decades, antimicrobial peptides (AMPs) have been investigated as a potential treatment for those infections, directly through their lytic effect or indirectly, due to their ability to modulate the immune system. There are still concerns regarding the use of such molecules in the treatment of infections, such as cell toxicity and host factors that lead to peptide inhibition. To overcome these limitations, different approaches like peptide modification to reduce toxicity and peptide combinations to improve therapeutic efficacy are being tested. Human defense peptides consist of an important part of the innate immune system, against a myriad of potential aggressors, which have in turn developed different ways to overcome the AMPs microbicidal activities. Since the antimicrobial activity of AMPs vary between Gram-positive and Gram-negative species, so do the bacterial resistance arsenal. This review discusses the mechanisms exploited by Gram-positive bacteria to circumvent killing by antimicrobial peptides. Specifically, the most clinically relevant genera, *Streptococcus spp.*, *Staphylococcus spp., Enterococcus spp.* and Gram-positive bacilli, have been explored.

## Introduction

Antimicrobial peptides, also known as host defense peptides (HDPs), are found in most life forms, being part of the innate immune system against pathogenic bacteria, fungi, parasites and viruses (Zasloff, 2002; Torrent et al., 2012). Due to the alarming increase in antimicrobial resistance to the commonly used drugs around the world and the lack in discovery of new drugs and alternative treatments, there is a growing concern among the scientific community that in a near future, the current clinical approaches might not be able to deal effectively with microbial infections. Therefore, AMPs have been suggested as an alternative therapeutic strategy, in combination or as a replacement for traditional antibiotics.

The development of antimicrobial resistance against AMPs is not as prevalent when compared to antibiotics, since AMPs targets are diverse and changes can interfere with the functionality of the cell, especially since the cell membrane is the main point of attack (Mahlapuu et al., 2016). However, bacteria can evolve quickly and grow resistant against AMPs *in vitro* (Andersson et al., 2016). Another approach in the use of AMPs is combination with traditional antibiotics, since both have shown to synergize, reducing microbial resistance (Moravej et al., 2018). A few AMPs have been translated into the clinic; polymyxins B, bacitracin, gramicidin S, daptomycin and vancomycin have been used for treatment of several types of bacteria. However, a number of questions are yet to be answered, such as the toxicity and stability *in vivo* of many peptides, as thoroughly reviewed (Jenssen et al., 2006; Vaara, 2009; Yount and Yeaman, 2012). The contact with human cells, such as erythrocytes, was shown to inhibit the activity of AMPs (Starr et al., 2016). Furthermore, physiological conditions of the host can interfere with the effectiveness of these molecules, along with the peptide’s pharmacokinetics (Jenssen et al., 2006). Though these are significant challenges, AMPs remain an interesting strategy and still expanding field, as many studies have tried molecular engineering as an approach to solve the concerns cited above. One such example is the production of synthetic D-enantiomeric peptides to avoid proteolytic degradation (de la Fuente-Nunez et al., 2015). So far, over three thousand different peptides have been identified, distributed among six different kingdoms (animalia, archaea, bacteria, fungi, plantae, protist), according to the Antimicrobial Peptide Database (APD) (aps.unmc.edu/AP/) (Wang et al., 2016). In humans, over 130 peptides have been described, and while the vast majority has been tested as potential antimicrobial drugs, AMPs have a larger impact than just direct antimicrobial effects, actively engaging with the host immune system, modulating its activity, promoting chemotaxis and cell recruitment, meddling with the inflammatory and wound healing pathways, among many different functions (Hancock et al., 2016; Mahlapuu et al., 2016; Haney et al., 2017). AMPs were also shown to have an anticarcinogenic effect, as extensively reviewed (Wang, 2014; Hancock et al., 2016; Haney et al., 2017; Yavari et al., 2018; Wang et al., 2019; Kunda, 2020).

An important group of antimicrobial peptides is the cathelicidins. The human representant of this group is LL-37, a cationic, amphipathic peptide, composed by 37 amino acid residues. Its precursor, hCAP18, was first isolated in neutrophils (Cowland et al., 1995; Sørensen et al., 1997) but can also be found in other cells, such as keratinocytes and mast cells (Frohm et al., 1997; Di Nardo et al., 2003). After its cleavage by neutrophil proteases, the peptide acquires its functional form (Sørensen et al., 2001). LL-37 effects have been extensively investigated, and include direct antimicrobial activity and immune modulation (Fabisiak et al., 2016; Mahlapuu et al., 2016; Xhindoli et al., 2016; Haney et al., 2017; Chen et al., 2018; Moravej et al., 2018). Cathelicidins are also found in many vertebrates, including farm animals, birds, reptiles and fish (Kościuczuk et al., 2012). Indolicidin, a 13 amino acid peptide expressed in bovine neutrophils, has antimicrobial activity against Gram-positive and Gram-negative bacteria (van Harten et al., 2018).

Another class of cationic and amphipathic antimicrobial peptides is the defensins, which can be divided in three main groups: α-defensins, β-defensins and θ-defensins. In humans, only α- and β-defensins can be found, while θ-defensins are present exclusively in Old World primates (Nguyen et al., 2003). Among human α-defensins, there are six peptides expressed: Human Neutrophil Peptide (HNP) 1 through 4 and Human Defensins (HD) 5 and 6. α-defensins can be found in many different tissues such as the gastrointestinal and respiratory epithelia, female reproductive tract and blood cells (Hancock et al., 2016). These peptides display direct antimicrobial activities and immunomodulatory effects, including chemotaxis (Wang, 2014; Moravej et al., 2018; Xu and Lu, 2020). β-defensins are expressed mainly in epithelial cells but also in monocytes, macrophages and dendritic cells (Hancock et al., 2016) and have an important role regulating the host microbiome (Meade and O’Farrelly, 2018; Xu and Lu, 2020).

Human Lactoferrin (hLF) is an 80 kDa bilobal glycoprotein, present in bodily fluids and neutrophils, which acts in the transport of metal ions, especially ferric iron (Fe^3+^) (Vogel, 2012). hLF displays a bacteriostatic effect through iron chelation, decreasing the extracellular concentration of this ion available to the microorganism. Furthermore, the iron-free molecule, Apolactoferrin (ApoLF), is able to interact with microbial cellular membranes, undergoing subsequent proteolysis which results in release of smaller and more potent cationic peptides, especially those found in the N-terminal lobe: Lactoferricin (LFcin), Lactoferrampin (LFampin) and LF1-11 (Sinha et al., 2013).

Human lysozyme, also named N-acetylmuramide glycanhydrolase, is often cited as the first antimicrobial protein discovered and is extensively used in industry (Ercan and Demirci, 2016; Wu T. et al., 2019). Lysozyme is a 14 kDa enzyme that binds to cell wall peptidoglycans, cleaving the links between different sugars, thus inducing cell rupture (Nawrocki et al., 2014; Wang, 2014). Similarly, to lactoferrin, peptides derived from the cleavage of lysozyme exhibit antimicrobial activity against Gram-positive and Gram-negative bacteria (Ibrahim et al., 2001, 2011; Mine et al., 2004; Hunter et al., 2005; Carrillo et al., 2018).

**FIGURE 1:**
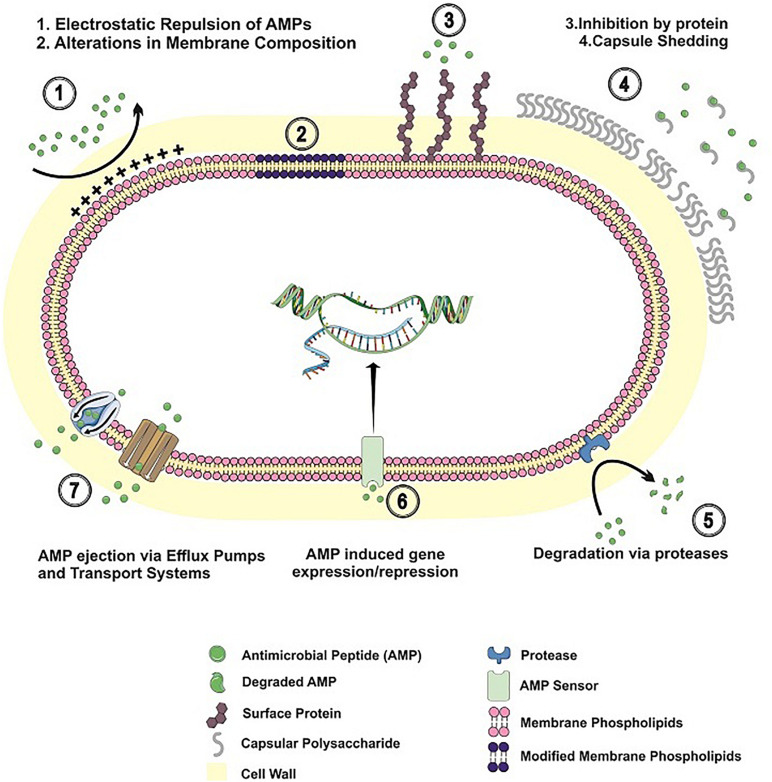
Resistance mechanisms to antimicrobial peptides in Gram-positive bacteria. AMP resistance mechanisms employed by Gram-positive bacteria are shown, including alterations in cell envelope charge/composition; AMP inhibition by binding to surface proteins/released capsular polysaccharide; AMP degradation by bacterial proteases; bacterial adaptation to AMP challenge; AMP extrusion by efflux pumps and transport systems.

With an array of antimicrobial peptides being produced by different human cells, bacteria have developed a number of strategies to prevent AMP binding, to avoid their lytic effects or to degrade the peptides, in order to thrive in the human host. In the next sections, the different mechanisms employed by Gram-positive bacteria to circumvent AMP action will be explored. Table 1 and Figure 1 summarize the resistance mechanisms employed by these bacteria.

**TABLE 1:** AMP resistance mechanisms in Gram-positive bacteria.

Resistance mechanisms	Species						References
*Modifications in membrane/cell wall structure*	Gram-positive bacilli	*Staphylococcus*	*Enterococcus*	GAS	GBS	Pneumococci	
D-alanylation of the membrane	X	X	X	X		X	Peschel et al., 1999; Poyart et al., 2001, 2003; Abachin et al., 2002; Frick et al., 2003; Cao and Helmann, 2004; Kristian et al., 2005; May et al., 2005; Fabretti et al., 2006; Fisher et al., 2006; Kovacs et al., 2006; Palumbo et al., 2006; Walter et al., 2007; Beiter et al., 2008; Abi Khattar et al., 2009; Cox et al., 2009; Jann et al., 2009; McBride and Sonenshein, 2011a; Saar-Dover et al., 2012; Simanski et al., 2013; Carvalho et al., 2015; Wydau-Dematteis et al., 2015; Kamar et al., 2017; Hirt et al., 2018.
Lysinylation of the membrane	X	X	X				Oku et al., 2004; Staubitz et al., 2004; Kraus and Peschel, 2006; Thedieck et al., 2006; Ernst et al., 2009; Samant et al., 2009; Bao et al., 2012; Shireen et al., 2013; Kumariya et al., 2015; Nasser et al., 2019.
O-acetylation of the peptidoglycan	X					X	Crisostomo et al., 2006; Pfeffer et al., 2006; Hebert et al., 2007; Aubry et al., 2011; Laaberki et al., 2011; Rae et al., 2011.
N-deacetylation of the peptidoglycan	X					X	Vollmer and Tomasz, 2000; Psylinakis et al., 2005; Boneca et al., 2007.
Glycosylation of the wall teichoic acids	X						Meireles et al., 2020.
Deacetylation of the N-acetylmuramic acid	X		X				Fukushima et al., 2005; Popowska et al., 2009; Benachour et al., 2012; Kobayashi et al., 2012; Grifoll-Romero et al., 2019.
Alterations in the membrane composition	X	X	X				Ming and Daeschel, 1993; Maisnier-Patin and Richard, 1996; Mazzotta and Montville, 1997; Verheul et al., 1997; Crandall and Montville, 1998; Dhawan et al., 1998; Bayer et al., 2000; Tsuda et al., 2002; Xue et al., 2005; Cremniter et al., 2006; Naghmouchi et al., 2006, 2007; Hachmann et al., 2011; Mishra et al., 2011, 2012; Kandaswamy et al., 2013.
Alterations in the transmembrane pH and potential	X						Bonnet et al., 2006.
Alterations in capsular polysaccharides						X	Beiter et al., 2008; Llobet et al., 2008; van der Windt et al., 2012; Geno et al., 2015; Kietzman et al., 2016; Bruce et al., 2018.
***Transport systems and efflux pumps***							
Transport systems	X		X	X	X	X	Manson et al., 2004; Matos et al., 2009; Majchrzykiewicz et al., 2010; McBride and Sonenshein, 2011b; Suárez et al., 2013; Martinez et al., 2019; Rafei et al., 2020.
***AMP sequestration/inactivation***							
AMP sequestration		X			X		Braff et al., 2007; Maisey et al., 2008.
Inactivation		X				X	Jin et al., 2004; Ren et al., 2004; Braff et al., 2007; Mukerji et al., 2012.
***Proteases and other proteins***							
Inactivation/degradation		X	X	X			Schmidtchen et al., 2002; Sieprawska-Lupa et al., 2004; Sedgley et al., 2009; Nesuta et al., 2017; Hirt et al., 2018.
Inhibitory molecules					X		Porta et al., 2019.
***AMP induced gene expression/repression***							
Sigma factors	X		X				Robichon et al., 1997; Dalet et al., 2000; Palmer et al., 2009; Le Jeune et al., 2010; Guariglia-Oropeza and Helmann, 2011; Ho et al., 2011.
Regulators	X	X	X		X	X	Dunman et al., 2001; Mascher et al., 2004; Mandin et al., 2005; Hyyryläinen et al., 2007; Meehl et al., 2007; Neoh et al., 2008; Dawid et al., 2009; Hachmann et al., 2009; Pietiäinen et al., 2009; Arias et al., 2011; Ho and Ellermeier, 2011; Boone and Tyrrell, 2012; Monniot et al., 2012; Yang et al., 2012, 2013, 2018; Bergholz et al., 2013; Khosa et al., 2013, 2016; Shaaly et al., 2013; Patel and Golemi-Kotra, 2015; Reyes et al., 2015; Wang et al., 2017; Xu and Lu, 2020.
Transcriptome/proteome alterations	X					X	Majchrzykiewicz et al., 2010; McQuade et al., 2012; Mucke et al., 2020.
Mannose phosphotransferase (Man-PTS) pathway	X		X				Ramnath et al., 2000; Héchard et al., 2001; Gravesen et al., 2002a,b; Vadyvaloo et al., 2004a,b; Diep et al., 2007; Tessema et al., 2009; Opsata et al., 2010; Kjos et al., 2011; Geldart and Kaznessis, 2017; Wu X. et al., 2019; Zasloff, 2002.
Cell sensors		X			X	X	Hamilton et al., 2006; Jones et al., 2007; Li et al., 2007a,b; Yung and Murphy, 2012; Tran et al., 2013; Joo and Otto, 2015; Khan et al., 2019; Martínez-García et al., 2019.

## AMP Resistance Mechanisms in Pathogenic Gram-Positive Bacteria

### Gram-Positive Bacilli

Gram-positive bacilli include some pathogenic, anaerobic spore-forming species, such as *Clostridium spp.*, *Listeria monocytogenes*, *Bacillus anthracis*, and *Bacillus cereus* (Chukwu et al., 2016; Schlech, 2019).

The genus *Clostridium* is composed of about 15 pathogenic species, of which the most common are *Clostridium difficile, Clostridium perfringens, Clostridium tetani*, and *Clostridium botulinum*. Although these species are similar, the pathologies caused by them are diverse (Fisher et al., 2005). *C. tetani* produces the tetanus neurotoxin (TeNT) that causes neurological disease (tetanus), characterized by muscle spasms and spastic paralysis of the limb muscles (Chapeton-Montes et al., 2019). *C. botulinum* produces the potent botulinum neurotoxin that causes a serious and fatal neuro-paralytic disease in humans and animals (botulism) (Brunt et al., 2018). *C. difficile* is the main causative agent of nosocomial diarrhea and gastroenteritis, which can lead to the development of asymptomatic or symptomatic diseases. Infection by *C. difficile* (ICD) has been increasingly reported in the United States (Lessa et al., 2015; Crobach et al., 2020). *C. perfringens* can also cause acute diarrhea, with an estimated death toll of 200,000 each year in Nigeria according to The World Health Organization (WHO) (Fisher et al., 2005; Chukwu et al., 2016).

*Listeria monocytogenes* is a foodborne pathogen that causes gastroenteritis in immunocompromised individuals, children, pregnant women and the elderly (Schlech, 2019). *L. monocytogenes* outbreaks in South Africa have reported around 1000 confirmed cases and 200 deaths in 2017–2018; in the United States, the bacterium was the causative agent in 147 confirmed cases and 33 deaths, making it the third most expensive foodborne pathogen in 2010, after *C. botulinum* (de Noordhout et al., 2014; Desai et al., 2019).

*B. anthracis* is the causative agent of anthrax and can manifest in four ways, namely: cutaneous, inhalation, gastrointestinal or injectable (Hagan et al., 2018; Chen et al., 2020). *B. cereus* causes foodborne diseases, such as gastrointestinal, diarrhea and emesis (Yu et al., 2019; Huang et al., 2020). In 2016, the European Union (EU) reported about 413 food-borne outbreaks caused by *Bacillus* toxins that affected 6657 people, ranking it the second most common cause of food-borne outbreaks in that year (Fiedler et al., 2019).

#### Mechanisms of AMP Resistance in Gram-Positive Bacilli

##### Modifications in membrane/cell wall structure

Since one of the most important mechanisms of AMP-based killing is the interaction with the negatively charged membrane, changing the membrane composition is a strategy used by many bacteria to survive AMPs action. Among those changes, the insertion of D-alanine in the lipoteichoic acids, a process named D-alanylation, is used to reduce the negative membrane charge, thus inhibiting interaction with AMPs. This resistance mechanism is regulated by the *dlt* operon, and it has been described in several *Bacillus* species, such as *B. cereus* (Abi Khattar et al., 2009), *B. anthracis* (Fisher et al., 2006), *B. thuringiensis* (Kamar et al., 2017) and *B. subtilis* (Cao and Helmann, 2004; May et al., 2005), *L. monocytogenes* (Abachin et al., 2002; Carvalho et al., 2015), *C. difficile* (McBride and Sonenshein, 2011a), *C. butyricum* (Wydau-Dematteis et al., 2015), *Lactobacillus plantarum* (Palumbo et al., 2006), and *Lactobacillus reuteri* (Walter et al., 2007).

Another mechanism of envelope modification is called lysinylation of the membrane; it consists of addition of L-lysine to the phosphatidylglycerol. A protein called MprF is essential for membrane lysinylation. In *B. anthracis*, a strain deficient in MprF was more susceptible to LL-37 and HNP-1 when compared with the wild type strain (Samant et al., 2009). In *L. monocytogenes*, MprF was shown to be essential in protection against gallidermin, HNP-1 and HNP-2 (Thedieck et al., 2006).

Bacteria can modify cell wall components, such as the peptidoglycan. O-acetylation of the peptidoglycan is able to reduce killing by lysozyme in *L. monocytogenes* and *B. anthracis* (Aubry et al., 2011; Laaberki et al., 2011; Rae et al., 2011). Modifications on cell wall constituents also include the N-deacetylation of the peptidoglycan and the glycosylation of the wall teichoic acids, which in *L. monocytogenes* and *B. cereus* (Psylinakis et al., 2005) is crucial to protect against lysozyme (Boneca et al., 2007), LL-37 and CRAMP, a cathelicidins found in mice (Meireles et al., 2020).

In *B. subtilis*, deacetylation of the N-acetylmuramic acid by the protein PdaC confers resistance against lysozyme attack (Fukushima et al., 2005; Kobayashi et al., 2012; Grifoll-Romero et al., 2019). Another protein, PdgA described in *L. monocytogenes* is responsible for a similar resistance mechanism inducing N-acetylation of the peptidoglycan; a study by Popowska et al. (2009) showed that a *L. monocytogenes* strain lacking PdgA was more susceptible to lysozyme and mutanolysin.

Changes in lipid composition are able to interfere with AMPs action, as shown for *L. monocytogenes*, where different proportions of lipids are found in bacteriocin resistant strains (Ming and Daeschel, 1993; Mazzotta and Montville, 1997; Verheul et al., 1997; Crandall and Montville, 1998; Naghmouchi et al., 2006, 2007). In *Listeria innocua*, changes in the proton motive force, via FoF1 ATPase, which altered the membrane potential were related with resistance to nisin (Maisnier-Patin and Richard, 1996; Bonnet et al., 2006).

A *B. subtilis* mutant resistant to daptomycin presents an irregular and more cationic membrane than the wild type, due to mutations in *pgsA* gene. The PgsA protein is responsible for the addition of phosphatidylglycerol to the membrane; in that sense, the diminished phosphatidylglycerol synthase function in the mutant strain was responsible for the increased resistance to daptomycin (Hachmann et al., 2011).

##### Transport systems and efflux pumps

A strategy employed by many bacterial species to evade antimicrobial host defense is by expelling the molecules using efflux pumps or ABC transporters. The same mechanism has been implicated in AMP expulsion (Bernard et al., 2007; Collins et al., 2010; McBride and Sonenshein, 2011b).

Subtilin is an antibiotic produced by *B. subtilis*; to avoid self-destruction, the bacterium possess an ABC transporter called SpaIFEG, this transporter ejects the subtilin to the extracellular environment (Stein et al., 2005). *B. licheniformis* is capable of producing bacitracin, an antibacterial peptide also produced by other *Bacilli*; similarly, to *B. subtilis*, the bacterium is immune to the antimicrobial due to the action of the BcrABC transporter, which ejects the AMP before it affects the producer cell (Podlesek et al., 1995; Ohki et al., 2003b).

In *C. difficile*, the *cpr* operon is responsible for the extracellular transport of peptides (McBride and Sonenshein, 2011b; Suárez et al., 2013). A similar ABC transporter, AnrAB, is also found in *L. monocytogenes*, able to export AMPs and antimicrobials, hence hindering their efficiency (Collins et al., 2010). *B. subtilis* also has similar detoxification systems, BceAB-RS, PsdRS-AB (also named Yvc-PQ-RS) and YxdJK-LM. These transporters are important for resistance and cell wall stress signaling against AMPs and antimicrobial drugs, such as bacitracin and lantibiotics (Mascher et al., 2003; Ohki et al., 2003a; Bernard et al., 2007; Rietkotter et al., 2008; Dintner et al., 2011; Staron et al., 2011; Kallenberg et al., 2013). The YtsCD ABC transporter, independently or in association with YwoA, is responsible for bacitracin resistance in *B. subtilis* (Bernard et al., 2003).

##### AMP induced gene expression/repression

Cell wall signaling can trigger the expression of many resistance-related genes such as sigma (σ) factors and global regulators in bacteria. In *L. monocytogenes*, sigma factors σ^B^ and σ^L^ and regulators such as VirR and LiaR regulate the expression of many virulence genes, such as the *dlt* operon, MprF – a protein responsible for adding L-lysine to membrane phospholipids–and ABC transporters, contributing to antimicrobial resistance (Mandin et al., 2005; Palmer et al., 2009; Samant et al., 2009; Bergholz et al., 2013). Lia-related regulators are also present in *B. subtilis*; in the presence of peptides that target the cell envelope, the stress sensor is activated and induces the expression of resistance genes such as LiaRS and other membrane modification genes (Mascher et al., 2004; Jordan et al., 2006; Hyyryläinen et al., 2007; Hachmann et al., 2009).

*Clostridium difficile* gene expression is also altered in the presence of AMPs. LL-37 induces overexpression of genes related to crucial functions; including those involved with cell wall and envelope homeostasis, ABC transporters and lysine metabolism (McQuade et al., 2012); similarly, bacitracin and lysozyme can alter the expression of extracellular σ factors (Ho and Ellermeier, 2011).

The mannose phosphotransferase (Man-PTS) pathway is an important resistance mechanism against bacteriocins. In *L. monocytogenes*, the activation of the Man-PTS pathway led to changes in metabolism, alteration of the membrane charge and addition of alanine in teichoic acids in strains resistant to class IIa bacteriocin (Ramnath et al., 2000; Gravesen et al., 2002a,b; Vadyvaloo et al., 2004a,b; Tessema et al., 2009; Wu X. et al., 2019). Although the Man-PTS pathway is a target for bacteriocins, in *Lactococcus lactis* and *Lactococcus garvieae*, it was shown to participate in resistance mechanisms, specifically in combination with LciA (Diep et al., 2007; Kjos et al., 2011; Daba et al., 2018; Tymoszewska et al., 2018). This pathway is also involved in bacteriocin resistance in *L. plantarum*, *Leuconostoc mesenteroides*, *Lactobacillus salivarius*, and *Lactobacillus acidophilus*, in combination with PedB, a protein that provides protection against the bacteriocin pediocin PA-1, in a complex, being able to avoid cell lysis by this AMP (Zhou et al., 2016). Similarly, in *Listeria innocua*, overexpression of *pedB* generated a more resistant phenotype (Monniot et al., 2012). In *L. innocua*, Man-PTS is regulated by a transcriptional activator (*lin0142*); inactivation of *lin0142* is related to resistance to pediocin (Xue et al., 2005).

The *B. subtilis* sigma factor ^V^ (*sigV*) is activated in presence of Lysozyme, regulating important resistance genes such as *oatA*, *dltABCD*, and *pbpX*, promoting protection by virtue of membrane alterations (Guariglia-Oropeza and Helmann, 2011; Ho et al., 2011). The alternative sigma factor 54 (*rpoN*) is relevant in mesentericin Y105 resistance in *Listeria monocytogenes*; strains lacking the monocistronic unit of *rpoN* showed a higher susceptibility to this AMP - a phenotype reverted after complementation - indicating that the resistance genes are under regulation of *rpoN* (Robichon et al., 1997).

### Staphylococci

The genus *Staphylococcus* is responsible for various infections in humans like impetigo, scalded skin syndrome, toxic shock syndrome, pneumonia, endocarditis, urinary tract infections, and many others. The most clinically relevant members of this genus are *Staphylococcus aureus*, *Staphylococcus epidermidis*, and *Staphylococcus saprophyticus*. They are grape-shaped, catalase producing Gram-positive spherical cocci. *S. aureus* are classified as coagulase positive, while *S. epidermidis* and *S. saprophyticus* do not show coagulase activity. Another trait shared by many staphylococci species is the presence of a carotenoid pigment called staphyloxanthin, which gives the colonies a golden color and has an inhibitory role against microbicide molecules and reactive oxygen species (ROS). Among their antigenic structures are the protein A, which binds to Fc region of immunoglobulin G (IgG) and prevents complement activation; the teichoic acids, which modulate mucosal adhesion and induce toxic shock through release of interleukin 1 (IL-1) and tumor necrosis factor (TNF); and polysaccharide capsule, with 11 different serotypes. Defense against AMPs in *Staphylococcus spp*.

#### Mechanisms of AMP Resistance in Staphylococci

##### Modifications in membrane/cell wall structure

*Staphylococcus aureus* is able to prevent AMP-mediated killing through modifications of the phosphatidylglycerol in the bacterial membrane by the multiple peptide resistance factor protein (MprF). The protein promotes the reaction of phosphatidylglycerol with lysin, generating lysylphosphatidylglycerol (Lys-PG), which is then translocated to the outer leaflet of the membrane (Oku et al., 2004; Staubitz et al., 2004; Ernst et al., 2009; Nasser et al., 2019). This results in a shift in membrane charge, and a subsequent repulsion of cationic AMPs.

The enhanced synthesis of the cationic phospholipid Lys-PG promotes changes in membrane fluidity also associated with increased resistance against different classes of AMPs in staphylococci. A study investigating the development of bacterial resistance to antimicrobial peptides demonstrated that exposure of *S. aureus* cultures to sub-lethal concentrations of magainin 2 and gramicidin D over several passages *in vitro* promoted resistance to these AMPs. The bacterial membrane adaptations induced by AMP exposure included an increase in net charge and altered membrane rigidity (Shireen et al., 2013).

Similarly, resistance to platelet microbicidal proteins (PMPs) in *S. aureus* has been linked with adaptations affecting membrane fluidity. A study investigating the mechanisms underlying *S. aureus* susceptibility to thrombin-induced PMP (tPMP-1) demonstrated that mutant strains with increased resistance to this AMP (either naturally occurring or artificially generated) displayed a high content of unsaturated lipids with longer chains (Bayer et al., 2000), which led to an enhanced membrane fluidity. Interestingly, tPMP resistance in *S. aureus* correlated with an increased virulence in both human and experimental endocarditis (Dhawan et al., 1998), highlighting the importance of this AMP in controlling *S. aureus* infection.

Resistance to cationic AMPs has also been associated with modifications in the cell wall teichoic acid by esterification with D-alanine, through the *dlt* operon, which reduces the net negative charge of the molecule. In *S. aureus* and *S. xylosus*, deletions of parts of the *dlt* operon induced a higher sensitivity to a variety of AMPs when compared to wild type strains. Interestingly, the increased susceptibility of the mutant strains were limited to cationic peptides, suggesting that electrostatic repulsion may be involved in resistance to cAMPs in *S. aureus* (Peschel et al., 1999; Jann et al., 2009; Simanski et al., 2013).

The presence of carotenoid pigments is another described mechanism of AMP resistance in staphylococci. These molecules are depicted as virulence factors, for their protective role against oxidative host defense mechanisms (Clauditz et al., 2006). Evidence suggests staphylococcal carotenoids can also provide protection against different antimicrobial peptides, through their effect on cell membrane stability (Mishra et al., 2011). In that work, a mutant strain with a defect in staphyloxanthin synthesis was compared with its supplemented counterpart in terms of susceptibility to a range of antimicrobial agents, including human HNP-1, PMPs, and polymyxin B. The supplemented strain showed a reduced susceptibility to the AMPs, which in this case was linked to a higher rigidity in the cell membrane. This apparent contrast with previous work showing a positive correlation between membrane fluidity and AMP resistance (Bayer et al., 2000) evidences the intricate balance driving peptide-cell membrane interactions. In that sense, extremes in rigidity or fluidity may hinder AMP insertion in the bacterial membrane.

##### AMP sequestration/inactivation

Another mechanism of *S. aureus* evasion from AMPs is trapping them by surface or secreted proteins and polysaccharides. *S. aureus* secrets a plasminogen activating protein, staphylokinase (SK), which converts it into plasmin. High concentrations of plasmin on the bacterial surface promote fibrinolysis, favoring tissue invasion and dissemination (Braff et al., 2007). It has been shown that SK can bind to and inactivate mCRAMPs (cathelicidin murine antimicrobial peptides) and α-defensins secreted by neutrophils, including HNP 1-3 (Jin et al., 2004; Braff et al., 2007) reducing the activity of AMPs in 80%. In an *in vivo* trial with mice, *S. aureus* strains expressing SK were more resistant to α-defensin. Similarly, addition of purified SK was able to increase survival of strains that did not produce this protein in presence of α-defensin, *in vitro* (Jin et al., 2004).

*Staphylococcus epidermidis* synthesizes the exopolysaccharide intercellular adhesin (PIA), a positively charged polymer of the extracellular matrix in biofilms, which can promote hemagglutination. Studies using mutant strains lacking this polysaccharide have shown a role for PIA in resistance to LL-37 and β-defensin (HBD-3) (Vuong et al., 2004a,b; Kocianova et al., 2005). The mechanism responsible for PIA-mediated protection against AMPs seems to involve electrostatic repulsion, since the lytic activities of these antimicrobial peptides are dependent on the salt concentrations (Vuong et al., 2004b). Besides the protective effect against AMPs produced in the skin, PIA can also limit destruction of *Staphylococcus* by neutrophils, by forming a mechanical barrier that prevents bacterial uptake by phagocytes (Vuong et al., 2004b).

Additionally, *S. aureus* is able to sequester iron from the heme site of hemoglobin through the Iron-regulated surface determinant (Isd), which is then released into the cytoplasm for metabolization (Foster et al., 2014). This ability is responsible for the bacterial resistance to the bacteriostatic effects of lactoferrin and other iron-binding peptides. Furthermore, resistance to the bactericidal action of lactoferricin can be induced in *S. aureus* by growing the bacterium in increasing peptide concentrations–which also promoted cross-resistance to other antimicrobials, like indolicidin and penicillin G (Samuelsen et al., 2005).

##### Proteases and other proteins

Antimicrobial peptides are relatively resistant to bacterial surface or secreted proteases, yet some proteases can cleave a broad spectrum of AMPs; one such example is aureolysin, which inactivates LL-37 by cleaving peptide bonds in its C-terminus, between residues Arg_19_-Ile_20_, Arg_23_-Ile_24_, and Leu_31_-Val_32_ (Sieprawska-Lupa et al., 2004). Thus, Aureolysin expression allows a higher survival in environments with high concentrations of LL-37 such as the phagolysosomes in macrophages and neutrophils.

##### AMP induced gene expression/repression

*Staphylococcus aureus* displays a phenotype known as small colony variant (SCV), which has been associated with persistent skin infections (Glaser et al., 2014). This phenotypic change allows *S. aureus* strains to evade innate immune responses, one of those being AMPs, since in SCVs, a higher MIC was observed (von Eiff et al., 2006; Garcia et al., 2013).

Analysis of four different AMPs found on the skin (beta-defensin – hBD-2 and -3, RNase 7, and LL-37) showed that SCV were more resistant to AMPs when compared with the wild type strains (Glaser et al., 2014). Similarly, a mutant strain with a hemin biosynthesis gene deletion, *hemB*, displaying a SCV phenotype, was less susceptible to three of the four AMPs tested, when compared with its complemented mutant exhibiting normal phenotype (Glaser et al., 2014). These results suggest that phase variation may be a mechanism of bacterial resistance to AMPs. This effect could be attributed to differences in membrane charge in the SCV strains, as suggested by Sadowska et al. (2002), however, in that work, SCVs showed an increased resistance to only a fraction of the AMPs tested.

*Staphylococcus aureus* expresses an AMP recognition system named Antimicrobial Peptide Sensor; this system comprises a sensor histidine kinase (ApsS), a DNA-binding response regulator (ApsR) and ApsX, responsible for interacting with the AMP. The ApsRSX regulators are responsible for the regulation of important genes related to AMP resistance, such as *mprF*, *vraFG* and the *dlt* operon (Li et al., 2007a,b; Martínez-García et al., 2019). Aps from *S. epidermidis* has shown the ability to interact with a broad variety of AMPs; in contrast to *S. aureus* in which Aps are active over a more limited spectrum of peptides (Joo and Otto, 2015). The ApsS is a transmembrane protein with an extracellular group sensitive to AMPs, composed by nine amino acids with a negative charge, which binds to AMPs and rapidly inactivates them (Li et al., 2007b).

The GraRS regulators induce the expression of *mprF* and *dltABCD*, when activated together with *vraFG*, as a response to AMPs and glycopeptides, whereas mutant strains negative for *graRS* or *vraFG* were more susceptible to the peptides as the surface alterations generated as protection mechanisms were reduced (Meehl et al., 2007; Neoh et al., 2008; Yang et al., 2012).

The *agr* global transcriptional regulator induced a super expression of *dltD*, a member of the *dlt* operon (Dunman et al., 2001). Another regulator is the LytSR, a transmembrane electrical potential sensor (Patel and Golemi-Kotra, 2015). AMP cell wall damage is partially due to changes in membrane polarization; therefore, deletion of LytSR increased susceptibility to HNP-1 and tPMPs, Interestingly, no conformational changes were found in mutant cells membrane, indicating an alternative resistance pathway (Yang et al., 2013).

The VraTSR is a bacterial sensor which responds to stress; it is involved in *S. aureus* resistance do methicillin (MRSA) and other antimicrobials that target the cell wall (Boyle-Vavra et al., 2013; Lee et al., 2019). Exposure to AMPs activates operons VraSR e VraDE, leading to a change in the transcriptional profile with the repression of virulence and metabolism genes, and an induction of genes that regulate envelope homeostasis (Pietiäinen et al., 2009).

### Enterococcus

Enterococci are a group of Gram-positive cocci comprising more than 30 species, of which *E. faecalis* and *Enterococcus faecium* are the most clinically relevant (*Fiore et al*., *2019*). They can be found in several environments such as water, soil and food, and are able to colonize the gastrointestinal tract of different animals. Enterococci are a leading cause of nosocomial infections – including endocarditis, urinary tract infections and bacteremia–being responsible for 14% of hospital infections in the United States (Weiner et al., 2016). The problem is aggravated by the increased intrinsic resistance and tolerance exhibited by these bacteria against several commercial antimicrobial agents, including β–lactams such as cephalosporins, and vancomycin (Kristich et al., 2014). In addition, enterococci rapidly acquire resistance to many classes of antibiotics upon treatment, thus posing a great public health threat.

#### Mechanisms of AMP Resistance in Enterococci

##### Modifications in membrane/cell wall structure

Similarly, to many other species previously cited, enterococci reshape their cell envelope composition in response to AMPs (Cremniter et al., 2006; Mehla and Sood, 2011; Mishra et al., 2012; Kandaswamy et al., 2013), with lysinated phosphatidylglycerol (Kraus and Peschel, 2006; Bao et al., 2012; Kumariya et al., 2015), addition of D-alanine to teichoic acids via *dlt* (Fabretti et al., 2006; Hirt et al., 2018) or MprF (Bao et al., 2012), also, the *N*-acetylglucosamine deacetylase PdgA (EF1843) contributes to lysozyme resistance in *E. faecalis*, by promoting peptidoglycan deacetylation (Benachour et al., 2012).

Many Enterococci species are able to perform O-acetylation of the cell wall peptidoglycan (Pfeffer et al., 2006) a mechanism related with resistance to lysozyme (Hebert et al., 2007).

##### Proteases and other proteins

Proteases and inhibitors are found in *E. faecalis*, either degrading or binding to the peptide, preventing their lytic effects. Among the proteases, GelE and SerE, a gelatinase and a serine protease, respectively, are able to degrade LL-37, HYL-20 – an α-helical amphipathic analog of a natural AMP present in bees – and GL13K, a peptide found in human saliva (Schmidtchen et al., 2002; Sieprawska-Lupa et al., 2004; Sedgley et al., 2009; Nesuta et al., 2017; Hirt et al., 2018). In addition, extracellular dermatan sulfate – a product released from proteoglycans after the activity of extracellular proteinases – was able to inhibit the activity of HNP-1 on *E. faecalis* (Schmidtchen et al., 2001), representing an important virulence mechanism for this bacterium.

##### Transport systems and efflux pumps

The Bcr transporter family is related to bacitracin resistance and is found in many enterococci species (Matos et al., 2009). In *E. faecalis*, BcrABD is an ABC transporter expressed in the presence of bacitracin. It is regulated by BcrR, which is responsible for the extracellular pumping of the polypeptide (Manson et al., 2004). However, the BcrAB is not the only mechanism of bacitracin resistance in *E. faecalis*; other two-component regulatory systems and ABC transporters were also described (Gebhard et al., 2014). In *S. aureus*, LtnIFE is responsible for protection against lacticin. *E. faecium* possess homologs with similar function (Draper et al., 2009).

##### AMP induced gene expression/repression

In *E. faecalis* and *E. faecium*, the Man-PTS pathway is also related to resistance against bacteriocins, however, there are several implications in metabolic pathways which could hinder the host colonization (Héchard et al., 2001; Opsata et al., 2010; Geldart and Kaznessis, 2017). Undecaprenyl pyrophosphate phosphatase (UppP) is also related to bacitracin resistance in *E. faecium* by reducing the amount of substrate for bacitracin-mediated cell death (Shaaly et al., 2013). Another regulator crucial for successful host colonization is the sigma factor SigV, which is involved in resistance to lysozyme, but not to nisin (Le Jeune et al., 2010). *rpoN* is responsible for encoding the sigma factor 54 in *E. faecalis*, an important factor for bacteriocin resistance. Interestingly, sensibility to other AMPs did not change in absence of this sigma factor (Dalet et al., 2000).

Both *E. faecalis* and *E. faecium* share the LiaFSR stress-induced regulatory pathway. LiaFS is the homolog of VraTS from *S. aureus*. Strains lacking *liaR* showed higher sensitivity against daptomycin, and LL-37, HBD-3, nisin, gallidermin–a type A lantibiotic, the synthetic antimicrobial peptide RP-1, mersacidin–a type B lantibiotic and friulimicin, a cationic lipopeptide in *E. faecalis* (Reyes et al., 2015; Wang et al., 2017). The deletion of *liaF*, along with *gdpD*, promoted a similar increase in resistance against daptomycin (Arias et al., 2011). The *liaFSR* and related genes, such as *liaX*, a sensor that inhibits LiaFSR, are directly related to the cell envelope alterations in response to antimicrobials (Tran et al., 2013; Khan et al., 2019).

### Group A Streptococci

Group A Streptococci (GAS) includes bacterial species such as *Streptococcus pyogenes* and *Streptococcus mutans* (Gold et al., 1973; Bessen et al., 1996). These bacteria are beta-hemolytic cocci and known to cause several diseases in humans, including mild conditions like scarlet fever, impetigo, strep throat, caries and cellulitis, and more severe illnesses like necrotizing fasciitis (flesh eating disease) and toxic shock syndrome (TSS) (Kristian et al., 2005).

### Streptococcus pyogenes

*Streptococcus pyogenes* comprises the considerable majority of Group A Streptococci (GAS); it is a pathogen responsible for several human diseases such as pharyngitis, scarlet fever, toxic shock syndrome, pneumonia and others (Lauth et al., 2009). Recent studies have shown that GAS was able to resist the action of several human antimicrobial peptides such as cathelicidin, LL-37 and the α-defensin (HNP-1) (Kristian et al., 2005; Lauth et al., 2009; Rafei et al., 2020). The surface exposed M-protein is used to classify the bacterium into different serotypes (Bessen et al., 1996; Lauth et al., 2009). Lauth et al. (2009) have shown that the N-terminal portion of M-protein can interact with LL-37, preventing its action on the bacterium membrane.

### Streptococcus mutans

*Streptococcus mutans* is an important pathogen that colonizes the human oral cavity being the most important caries agent (Gold et al., 1973). Interestingly, several *S. mutans* strains have been described as resistant to salivary AMPs and bacitracin (Tsuda et al., 2002; Kitagawa et al., 2011; Tian et al., 2018).

A study by Phattarataratip et al. (2011) compared *S. mutans* strains isolated from 60 children divided into two groups (caries-free and caries-active) and they found that strains isolated from the caries-active group were significantly more resistant to salivary AMPs such as LL-37, α-defensins and β-defensins, in comparison to caries-free strains. Their analysis also correlates this resistance to an ecological advantage over the less resistant strains, which reinforces the importance of AMPs in controlling *S. mutans* colonization (Phattarataratip et al., 2011).

#### Mechanisms of AMP Resistance in Group A Streptococci

##### Modifications in membrane/cell wall structure

Since most AMPs present cationic nature, the negative charge of the bacterial surface is important for the bactericidal activity of these molecules. Kristian et al. (2005) showed that the D-alanylation (regulated by the *operon dlt* (DltABCD)) of *S. pyogenes* lipoteichoic acid is related with resistance to cationic AMPs, lysozyme and low pH, and it was also associated with an increased survival against neutrophil killing; this phenomenon is due to the increase of positive surface charge caused by the D-alanylation on the cell membrane. In another study, (Cox et al., 2009) using a knockout strain for the dltABCD operon found that the DltA mutant displayed a drastic reduction in the expression of M protein and SIC (Serum Inhibitor of Complement) (Frick et al., 2003), showing that the *operon dlt* (DltABCD), specifically the *dltA* gene regulates the expression of genes involved in AMP resistance.

A study published by Tsuda et al. (2002) investigated the mechanisms that allow *S. mutans* to resist bacitracin; they found that mutant strains lacking the *rgp* locus (a six gene operon) presented up to five times more sensibility to bacitracin than the wild type counterpart. A possible mechanism to explain this sensitiveness is the fact that the *rgp* locus is involved in the synthesis of rhamnose-glucose polysaccharide (RGP), a cell wall component; mutations affecting this process render the bacterium more sensitive to bacitracin (Yamashita et al., 1998).

##### Proteases and other proteins

*Streptococcus pyogenes* is able to limit LL-37 action through degradation by the cysteine proteinase, SpeB. In presence of the inhibitor E64 (which inhibited the cysteine proteinase) the bacterium’s ability to degrade LL-37 was hampered, making it more susceptible to this CAMP. This effect highlights the importance of proteinase SpeB in LL-37 degradation (Schmidtchen et al., 2002).

Similarly, to previously described for *Enterococci*, *S. pyogenes* secretes proteases that are able to cleave proteoglycans containing dermatan sulfate, releasing it to the extracellular space. The extracellular dermatan sulfate was able to neutralize neutrophil-derived alpha-defensin, protecting the bacteria from its bactericidal activity (Schmidtchen et al., 2002).

M-protein is the most studied protein in *S. pyogenes*; variations in M-protein sequence are used to classify the bacterium into different serotypes (Lauth et al., 2009). A study by Lauth et al. (2009), showed that the M protein type 1 protects the bacterium from killing by cathelicidins LL-37 (human) and mCRAMP (mouse). The proposed mechanism involves M1 binding to and trapping the cathelicidin before it can reach the cell wall. They also showed that this protection is type specific once M protein type 49 did not protects the bacterium the same extension of M1, moreover, they found that strains isolated from invasive diseases patients were more resistant to LL-37 action than the strains isolated from asymptomatic patients (Lauth et al., 2009).

Another strategy employed by *S. pyogenes* to resist AMP attack is the Serum Inhibitor of Complement (SIC). This protein was initially identified as a virulence factor protecting the bacterium against killing by the complement system membrane attack complex (Akesson et al., 1996). Further studies from the same group showed that SIC is important for bacterium full virulence, once it is able to bind to defensins and LL-37, protecting the bacterium against these molecules (Frick et al., 2003).

##### Transport systems and efflux pumps

*Streptococcus mutans* express the ABC transporter, *mbr*, an operon composed by 4 genes. Mutant strains that do not express the full transporter were 100 to 120-fold more sensitive to bacitracin than the wild type strain (Tsuda et al., 2002). A more recent study from the same group, analyzed the transcriptome of the bacterium after exposure to bacitracin. They found 8 genes (SMU.302, SMU.862, SMU.863, SMU.864, *mbrA, mbrB*, SMU.1479, SMU.1856c) that were upregulated upon AMP challenge; of those, the MbrC protein acts as a transcriptional regulator for MbrA and MbrB–which are part of the ABC transporter and are required for bacitracin resistance–and it also controls the expression of SMU.863 and SMU.864, also described as ABC transporters involved in bacitracin resistance by *S. mutans* (Kitagawa et al., 2011).

The *S. mutans bceABRS* operon encodes an ABC transporter (BceAB) and a two-component system BceRS. The entire four-component system was shown to be important for protection against bacitracin, defensins (α and β), LL-37 and histatin (Tian et al., 2018). In contrast with wild type *S. mutans*, mutant strains lacking each *bceABRS* gene failed to form biofilms in response to a sub-inhibitory concentration of β-defensin. This data suggest that BceABRS also acts as a sensor, promoting a switch to an AMP resistant phenotype upon challenge (Tian et al., 2018).

### Group B Streptococci

*Streptococcus agalactiae*, also referred to as Group B Streptococci (GBS), is an opportunistic pathogen that colonizes the gastrointestinal, genitourinary tracts and, in women, the vaginal mucosa. The biggest concern regarding infections with GBS is in pregnant women, because it can be transmitted vertically and results in serious neonatal consequences, causing several diseases to the newborn, such as meningitis, sepsis and pneumonia (Shabayek and Spellerberg, 2017).

The incidence of infections by *S. agalactiae* is twice as high in pregnant women when compared to non-pregnant women. Most GBS infections occur during labor, but there is also a chance of infection after delivery. In the United States, GBS infection rates range from 0.1 to 0.8 per 1,000 childbirths. Worldwide, the rates in pregnant women are 0.38 per 1,000 childbirths, with 0.2 in 1000 mortality rate (Raabe and Shane, 2019). GBS infection is also associated with an increased chance of premature delivery. Around the world, premature birth is an important contribution to the death of newborns; approximately 10% of deaths in neonates are caused by GBS infection (Vornhagen et al., 2017).

#### Mechanisms of AMP Resistance in Group B Streptococci

##### Modifications in membrane/cell wall structure

In *S. agalactiae*, the *dlt* operon is essential for resistance against AMPs. Deletion of *dltA* hinders bacterial survival ability *in vivo* and reduces the resistance to AMPs, possibly due to an increased interaction with the peptide. Interestingly, the D-alanylation of the membrane seems to induce resistance by enhancing cell envelope strength rather than the interference with the ionic charge of the membrane (Poyart et al., 2001, 2003; Saar-Dover et al., 2012).

##### Proteases and other proteins

*Streptococcus agalactiae* is intrinsically resistant to nisin via NSR or SaNSR, a nisin-specific enzyme that cleaves and hinders the activity of the peptide. It is expressed by the *nsr* operon with other lantibiotic resistance genes, such as nsrFP and nsrRK (Khosa et al., 2013, 2015, 2016). However, modified nisin molecules were able to maintain activity against strains possessing SaNSR (Hayes et al., 2019; Zaschke-Kriesche et al., 2019). Another mode of escaping the degrading activity of AMPs is via inhibitory molecules capable of binding to the nisin site of SaNSR (Porta et al., 2019). A phosphoglycerate kinase of GBS was also identified to participate in AMP resistance. Though the mechanism is unknown, it is supposed to include direct binding of the peptides (Boone and Tyrrell, 2012).

##### Transport systems and efflux pumps

NsrFP is an ABC transporter which exports nisin to the extracellular medium. The transporter binds to the N-terminal portion of the peptide and releases it, preventing cell death, even in absence of the two-component regulator NsrRK (Reiners et al., 2017).

In *S. sanguinis*, a study involving multiple gene screening reported a role for sag1003 in AMP resistance against nisin and bacitracin. The gene is predicted to be an efflux pump against AMPs and a transposon-induced mutagenesis caused a higher sensitivity against both AMPs in a plate-based minimum inhibitory concentration (MIC) assay (Boone and Tyrrell, 2012).

##### AMP sequestration and inactivation

*Streptococcus agalactiae* pili are important against host defense mechanisms, such as AMPs. The sequestration of AMPs by pili prevents the interaction with the membrane targets. Strains lacking *pilB*, one of the pilus subunit proteins, were more sensitive to AMPs and less virulent overall, supposedly by virtue of resistance against LL-37, mCRAMP and polymyxin B. Heterologous overexpression of PilB from *S. agalactiae* in *L. lactis* showed similar results (Maisey et al., 2008).

##### AMP induced gene expression/repression

The *bceRSAB* is a detoxification system in GBS, regulating the gene expression against AMPs, such as *dltA*, promoting resistance. Strains lacking the regulator BceR showed an increased susceptibility against bacitracin and LL-37 and reduced overall virulence (Yang et al., 2019).

The insertion of an inactivation transposon in *sag1003* induced a reduction of phosphoglycerate kinase in the cell wall (Boone and Tyrrell, 2012).

The two-component regulator NsrRK is responsible for the transcriptional control of the NSR pathway (*nsr* and *nsrFP*) in *L. lactis* strains capable of synthesizing nisin. In GBS, a very similar *nsr* operon was described, indicating the possibility of an analogous system (Khosa et al., 2013, 2016).

Hamilton et al. (2006) identified a surface-associated penicillin-binding protein called PBP1a, which is encoded by the *ponA* gene. A mutant Δ*ponA* strain was more susceptible to AMPs from cathelicidin and defensin families, but the exact mechanism involved in this protection is still unknown (Hamilton et al., 2006; Jones et al., 2007).

### Streptococcus pneumoniae

*Streptococcus pneumoniae* (pneumococcus) is responsible for around 1 million deaths worldwide every year, and an increasing drug resistance case reporting (Tramper-Stranders, 2018). It is the main causative agent in community acquired bacterial pneumonia, and it can also cause otitis media, conjunctivitis, sinusitis and more severe diseases like meningitis and bacteremia.

Pneumococci are frequent colonizers of the upper respiratory tract, and a single person may be colonized with multiple strains concomitantly for months. Asymptomatic carriers are also the main source of pneumococcal transmission (Khan and Pichichero, 2014). In this highly colonized niche, AMP resistance confers an important competitive advantage both inter and intra species. Pneumococci display a vast number of adaptations that promote increased AMP resistance, from envelope modifications to AMP sequestration, as described next.

#### Mechanisms of AMP Resistance in *S. pneumoniae*

##### Envelope modifications

A vast majority of clinically relevant pneumococcal isolates are covered by a thick polysaccharide capsule with variable structure, which protects the bacterium from host immune defenses. Based on their high immunogenicity and protective efficacy, capsular polysaccharides comprise the basis of the current pneumococcal vaccines, alone or in fusion with carrier proteins (Darrieux et al., 2015; Geno et al., 2015; Converso et al., 2020).

Variations in capsule polysaccharide () locus determine the classification of pneumococci in over 95 different serotypes. These include mainly negative structures, with a few being neutral or positive. Negatively charged free capsular polysaccharides (but not neutral or positive ones) have displayed a role in preventing AMP attack. These purified anionic CPS were able to increase the resistance of non-encapsulated mutant pneumococci to HNP-1 and polymyxin B, an effect that was abrogated when the CPSs lost their negative charge through reaction with polycations. One proposed mechanism is that exposure to antimicrobial peptides triggers CPS release, which trap the AMPs and shield the bacterium (Llobet et al., 2008). This capsule shedding has been demonstrated to occur *in vivo*, thus comprising a potential strategy to prevent AMP-mediated killing. Capsule shedding can be triggered by autolysin (LytA) activity, promoting bacterial resistance to LL-37 and favoring colonization (Kietzman et al., 2016).

Surface-attached capsular polysaccharides, on the other hand, have shown the opposite effect, rendering the bacteria more susceptible to AMP action, in comparison with non-encapsulated isogenic mutants (Beiter et al., 2008). This effect was observed with different capsular types, including CPS 2, 4, 9V and 19F, and the zwitterionic serotype 1. As shown for other Gram-positive bacteria, D-alanylation of teichoic acids in non-encapsulated pneumococci results in increased resistance against killing by neutrophil extracellular trap (NET)-derived components (Beiter et al., 2008). This effect is aided by surface proteins, like the choline binding protein LytA and PgdE, which contribute to reduce the surface negative charge (discussed further). In that sense, the presence of capsule could mask the underlying protective mechanisms against AMPs. This apparent detrimental effect of capsule production over pneumococcal sensitivity to AMPs is possibly overcome by the capsule shedding as previously discussed, and also by its ability to protect the bacterium against mucus and phagocytic cell repulsion (Geno et al., 2015). Furthermore, the effect may not be applicable to all capsular serotypes; great variations in carriage, invasiveness and prevalence exist among capsule types, which have been associated with variations in surface net charge (Li et al., 2013). In that sense, the investigation of AMP resistance in a higher number of pneumococcal serotypes may provide new insights into the role of surface CPS on AMP resistance. For instance, type 4 TIGR4 and its isogenic capsule-negative mutant have shown increased sensibility to CXCL10, LL-37, and nisin, when compared with the type 2 strain, D39 (Bruce et al., 2018).

Another study has shown that non-encapsulated pneumococci are more resistant to neutrophil proteases, elastase and cathepsin G–a feature that also contributes to the ability to colonize the nasopharynx (van der Windt et al., 2012).

Cell wall modifications by the *dlt* operon have also been shown to promote resistance against nisin and gallidermin in pneumococci, an effect that was consistent with an increased release of D-alanine upon hydrolysis in wild type versus *dltA*-negative mutant stains (Kovacs et al., 2006).

Pneumococci express two enzymes, PgdA and Adr, that modify peptidoglycans on the bacterial cell wall. PgdA is a N-acetylglucosamine deacetylase (Vollmer and Tomasz, 2000), while Adr is an O-acetyl transferase that acetylates muramic acid residues on the peptidoglycan backbone (Crisostomo et al., 2006). Double mutant strains unable to perform these modifications displayed lower ability to colonize lysozyme-sufficient mice, but behaved similarly, to wild type pneumococci in mice lacking lysozyme production. In contrast, mutants in only one of the molecules colonized mice more efficiently than the wild type strain, in both Lys-producing and Lys-deficient mice (Davis et al., 2008). Taken together, these results indicate that the ability to limit lysozyme attack by modifying the cell wall contributes to successful colonization of the host.

##### AMP sequestration/inactivation

Studies from our group and others have reported a role for pneumococcal surface protein A (PspA) in bacterial resistance to AMPs. PspA is an exposed virulence factor with structural and serological variability (Goulart et al., 2013; Converso et al., 2017b) that has been successfully evaluated as a vaccine candidate in different infection models (Darrieux et al., 2007; Goulart et al., 2013; Converso et al., 2017a, 2020). It prevents complement activation/deposition on the pneumococcal surface, limiting bacterial uptake by phagocytes (Ren et al., 2004; Mukerji et al., 2012).

Pneumococcal surface protein A can bind to and prevent the lytic action of lactoferrin (Hakansson et al., 2001; Shaper et al., 2004). Furthermore, anti-PspA antibodies induced by vaccination were able to enhance the bactericidal effect of apolactoferrin (the iron-free form of the molecule) by blocking PspA interaction with that protein (Shaper et al., 2004; Andre et al., 2015). This protective effect of PspA over pneumococci was diminished when lactoferrin was combined with lysozyme (Andre et al., 2015). This set of data suggests PspA is able to prevent the lytic action of cationic peptides against pneumococci, possibly by binding to these molecules through their active sites. This interaction has been demonstrated for lactoferrin (Senkovich et al., 2007).

Pneumococcal surface protein A has also been shown to interfere with the bactericidal activity of NETs (Martinez et al., 2019). Mutants lacking PspA were more susceptible to trapping by NETs, an effect that was dependent on PspA type. In addition, incubation with anti-PspA antibodies promoted NET formation (Martinez et al., 2019). Taken together, the data indicates that PspA is able to directly prevent killing by AMPs, and also to limit the bactericidal mechanisms of neutrophils.

##### Efflux pumps and transport systems

Pneumococci express and efflux pump, MefE/Mel, which confers resistance to macrolides. *mfE* expression is induced upon bacterial incubation in presence of LL-37. In consequence, pneumococci develop resistance to LL-37 and erythromycin *in vitro* (Zahner et al., 2010).

A second, MacAB-like efflux pump described in *S. pneumoniae*, comprised by the spr0693-spr0694-spr0695 operon, is also involved in resistance against antimicrobial peptides and antibiotics, like LL-37, nisin and bacitracin (Majchrzykiewicz et al., 2010; Yang et al., 2018).

The oligopeptide import ABC transport system Opp (AmiACDEF) has been implicated in resistance against CXCL10, a chemokine with antimicrobial activity against several pathogens (Yung and Murphy, 2012). In that work, mutant strains lacking the permease were less susceptible to CXCL10 and nisin, when compared with the parent D39 strain. Although the precise mechanism responsible for this effect is not fully understood, it is known that AmiA-F has additional pleiotropic roles in pneumococcal physiology, quorum sensing, and virulence (Bruce et al., 2018).

##### AMP induced gene expression/repression

Cell wall modifications in pneumococci can be triggered by AMPs. Treatment with lysozyme leads to upregulation of the *dlt* locus through the CiaRH sensoring system, resulting in lipoteichoic acid (LTA) modifications and increased inflammatory responses, which in that case contributed to bacterial shedding and transmission (Zafar et al., 2019). Thus, D-alanylation of the cell wall – a mechanism of AMP resistance shared among different Gram-positive microbes–can be induced, in pneumococci, by treatment with antimicrobial proteins that target the bacterial cell wall.

Incubation with LL-37 can also trigger an adaptive response in pneumococci. The transcriptome analysis of pneumococci treated with LL-37 revealed a profound effect on the bacterial genome, with 10% of the genes displaying an altered expression upon challenge (Majchrzykiewicz et al., 2010). The up-regulated genes included those involved in cell wall biosynthesis (dlt), bacteriocin production, virulence (such as the proteases HtrA e PrtA) and bacteriocin production, as well as transcriptional regulators and putative ABC transporters. Interestingly, the serino-protease HtrA is also involved in resistance against other environmental stressors, like high temperature and oxidative stress (Dawid et al., 2009). The choline binding protein PspA and LysM protein (SP 0107)–predicted to be involved in cell wall metabolism–were down regulated in presence of LL-37. Interestingly, LL-37 had a much more dramatic effect on pneumococcal gene expression patterns, when compared with bacterial-derived AMPs that act on the same bacterial targets (nisin and bacitracin). Furthermore, mutant strains lacking these genes revealed an increased susceptibility to treatment with LL-37, confirming the employment of multiple defense strategies against AMPs in pneumococci (Majchrzykiewicz et al., 2010). A more recent study evaluating the proteome of pneumococci treated with LL-37 has also reported a large number of proteins with altered abundance, including transporters, proteins involved in gene regulation and cell wall modification, virulence factors (such as Pht family) and the protease HtrA (Mucke et al., 2020). This result suggests that multiple mechanisms cooperate in pneumococcal response to AMPs.

##### AMP resistance as a competitive advantage

A study investigating the susceptibility of multiple pneumococcal isolates – both clinical and from carriage–to LL-37 and HNP-1 found great variations in AMP resistance, with no correlation with AMP or capsule type, although clinical isolates were, in general, more susceptible than were carriage isolates (Habets et al., 2012). Furthermore, the study reported that AMP challenge could affect bacterial fitness in competitive assays. This result suggests a role for AMPs in driving intraspecific competition among pneumococci in the nasopharynx, contributing the bacterial genetic diversity in this niche.

## Discussion

Antimicrobial peptides are central players in the innate immune defense against pathogenic bacteria. Unsurprisingly, microbes have developed several strategies to overcome AMP activity, which allow them to efficiently colonize/invade the host. The present review summarizes the strategies adopted by Gram-positive pathogenic bacteria to resist AMP action. Some of these mechanisms, like cell wall modifications, are shared by several pathogens, highlighting their pivotal contribution to bacterial survival within the host. Other factors such as surface proteins and virulence factors are microbe-specific, revealing a myriad of adaptations that comprise the bacterial arsenal against AMPs.

The alarming increase in antibiotic resistance has prompted the search for alternative treatment options. In this scenario, AMPs emerge as a promising strategy to control bacterial infections. This rationale is reinforced by the demonstration that antibiotic resistance in bacteria usually correlates with a collateral sensitivity to AMPs (Lazar et al., 2018).

Several approaches employing AMPs have been tested with encouraging results. The use of AMP combinations is of particular interest, since these molecules can potentiate each other’s action, and also improve the therapeutic efficacy of conventional antibiotics, through synergistic interactions (Reffuveille et al., 2014), This represents an excellent strategy to slow down or minimize bacterial resistance development. In that sense, a better comprehension of the mechanisms employed by bacteria to resist AMP action is pivotal for the development of more effective therapeutic strategies. Furthermore, since many bacterial molecules involved in AMP resistance are important virulence factors, the present review presents numerous potential targets for vaccine development, and also contributes to elucidate the mechanisms driving intra-and interspecies competition within the host.

## Author Contributions

LA, TC, BM, MC, NW, LN, MG, and MD drafted the manuscript. LA, TC, and MD revised the manuscript. LA and MC drafted the table. LA, MC, and MD produced the Figure. All authors read and approved the final manuscript. None of the authors have any competing interests.

## Conflict of Interest

The authors declare that the research was conducted in the absence of any commercial or financial relationships that could be construed as a potential conflict of interest.
